# Genetics of Familial Hypercholesterolemia: New Insights

**DOI:** 10.3389/fgene.2020.574474

**Published:** 2020-10-07

**Authors:** Michal Vrablik, Lukas Tichý, Tomas Freiberger, Vladimir Blaha, Martin Satny, Jaroslav A. Hubacek

**Affiliations:** ^1^3rd Department of Internal Medicine, 1st Faculty of Medicine, Charles University, Prague, Czechia; ^2^Centre of Molecular Biology and Gene Therapy, University Hospital, Brno, Czechia; ^3^Centre for Cardiovascular Surgery and Transplantation, Brno, and Faculty of Medicine, Masaryk University, Brno, Czechia; ^4^Internal Gerontometabolic Department, Charles University and University Hospital Hradec Kralove, Hradec Kralove, Czechia; ^5^Experimental Medicine Centre, Institute for Clinical and Experimental Medicine, Prague, Czechia

**Keywords:** familial hypercholesterolemia, gene score, epidemiology, variants, polygenic FH

## Abstract

Familial hypercholesterolemia (FH) is one of the most common monogenic diseases, leading to an increased risk of premature atherosclerosis and its cardiovascular complications due to its effect on plasma cholesterol levels. Variants of three genes (*LDL-R*, *APOB* and *PCSK9*) are the major causes of FH, but in some probands, the FH phenotype is associated with variants of other genes. Alternatively, the typical clinical picture of FH can result from the accumulation of common cholesterol-increasing alleles (polygenic FH). Although the Czech Republic is one of the most successful countries with respect to FH detection, approximately 80% of FH patients remain undiagnosed. The opportunities for international collaboration and experience sharing within international programs (e.g., EAS FHSC, ScreenPro FH, etc.) will improve the detection of FH patients in the future and enable even more accessible and accurate genetic diagnostics.

## Historical Introduction

Variants of the genes causing familial hypercholesterolemia (FH) have recently been shown to be important risk factors leading to premature atherosclerotic cardiovascular disease (ASCVD) and premature death. However, these straightforward associations have not always been considered unambiguously evidenced and have at times been rejected.

A small but interesting study by Sijbrands et al. (2001) suggested that mortality in subjects with FH (three large pedigrees, carriers of the Val408Met variant, a total of 412 subjects through 8 generations) significantly varied over time. In the nineteenth century, the mortality of FH subjects was lower than that in the general population. The peak mortality in FH patients (twofold increased risk of premature CVD death) was reached by the first half of the last century followed by a decreasing trend thereafter.

To a certain extent, this observation can be explained by the fact that in the 19th century, life expectancy was much lower and mortality causes were dominated by infections (Zaffiri et al., 2012). In contrast, in the twenty-first century, non-communicable diseases top mortality statistics as the most frequent causes of death^1^, and it has been shown that plasma cholesterol plays an important role in protection against bacterial infection. One of the most robust pieces of evidence indicates an increased risk of severe infections (sepsis) in subjects with low LDL-C (Guirgis et al., 2016).

Nonetheless, with the current progress in the treatment of infections (antibiotics), a sedentary lifestyle together with abundant caloric intake lead to an environment (for review see Loscalso, 2004; Hubacek, 2009) in which FH causing variants show their detrimental effects, increasing the risk of ASCVD.

## Familial Hypercholesterolemia

### Definition of Familial Hypercholesterolemia

Familial hypercholesterolemia (FH; OMIM ID-143890) is an autosomal dominant inherited disease, the cause of which is most often a variant in the gene for the LDL receptor (*LDLR*), less often a variant in the gene for its ligand, apolipoprotein B 100 (*APOB*). Rarely, a specific gain-of-function type of variant in the subtilisin-kexin type 9 proprotein convertase gene (*PCSK9*) may be the cause. The phenotypic expression of FH can also be caused by variants in the LDL-receptor adapter protein (*LDLRAP*) gene with a recessive inheritance. As a result of the abovementioned variants, the removal of low-density lipoproteins (LDL) from the blood is significantly slowed down in hepatocytes. Consequently, the level of LDL cholesterol (LDL-C) (and thus total cholesterol as well) increases very substantially: in patients with heterozygous FH, total cholesterol is above 8 mmol/l (usually approximately 9–12 mmol/l) and LDL-C is above 5 mmol/l (usually 6–9 mmol/l) but may be even higher (Cuchel et al., 2014). Both HDL cholesterol and triglyceride levels are usually normal, but hypertriglyceridemia does not rule out FH. The rare homozygous form of FH presents with LDL-C levels above 13 mmol/l without therapy or persistent LDL-C elevations above 8 mmol/l with statin therapy (Cuchel et al., 2014). However, blood lipid levels can have considerable variability, depending especially on the type of variant but also on lifestyle or other associated diseases, which makes it more difficult to diagnose FH, suggesting that the disease should always be considered. It has been well established that very high cumulative LDL-C levels significantly accelerate the development of atherosclerosis, and because patients with FH are exposed to markedly increased cholesterol levels for most of their lives, cardiovascular disease (especially myocardial infarction) may manifest at a very early age (in the first decade in untreated homozygotes, after the second decade in severe FH heterozygotes). More than 1,700 different variants have been described in the LDL receptor gene that affect the structure and function of the LDL receptor in various ways; consequently, the level of LDL-C in patients with FH caused by a variant in this gene can have considerable variability (Leigh et al., 2017). In contrast, only a few variants are described in the apolipoprotein B gene, with the vast majority of patients having a single type of variant and thus relatively uniform LDL-C levels (and on average lower than in the LDL-receptor gene). A causal variant in the gene for PCSK9, which may lead to a clinical picture of FH, has so far been identified in only one proband in the Czech Republic and is therefore a rare cause of FH. The practical conclusion of the dominant genetic transmission of FH is the fact that a patient with confirmed FH always has at least one parent with the same disease, and their offspring or siblings have a 50% risk of inheriting FH. Therefore, the so-called cascade screening in families, in which genetic testing of a known variant is performed in relatives of an already diagnosed patient, appears to be a suitable method for searching for people with FH.

### Epidemiology of FH

The classic work of Goldstein et al. (1982) from the 1990s reported a 1:500 prevalence of heterozygous FH. However, a 2012 Danish study of a large sample of more than 69,000 people showed a much higher prevalence, approximately 1:250, of heterozygous FH; similar data have been replicated in other European populations (Benn et al., 2012). It can be assumed that the Czech population will have the same disease frequency as other white populations. These new findings indicate that FH is the most common congenital metabolic disorder in humans. The homozygous form of FH is rare. Its incidence has so far been estimated at 1:1,000,000; the abovementioned recent studies estimate the prevalence of homozygous FH at 1: 160,000–1:300,000 (Hu et al., 2020).

### Methodological Considerations in FH Genetic Testing

A large array of methods has been used for analysis in patients with the FH phenotype to identify underlying genetic defects to date. All of them have their own advantages and disadvantages. The cost of genetic testing has dramatically fallen over the past decade due to major advances in sequencing technology, especially the introduction of next-generation sequencing (NGS).

This technology offers the possibility of sequencing the whole human genome or exome (Farhan and Hegele, 2014) in a relatively short time and produces a large amount of genetic data. Given the vast amount of data generated, it is critical to have an integrated and validated bioinformatics pipeline, which assembles millions of overlapping small sequenced fragments into a string of large contiguous sequence information. Not all the data are useful for routine diagnostics, of course, especially regarding the focused diagnosis of a specific condition such as FH. This fact led to the development of a gene panel that produces more relevant data with an enhanced likelihood of detecting potentially clinically useful variants (Hegele et al., 2015).

This technology can also be used for the analysis of a high number of SNPs, which have been described in connection with the construction of population-specific/unspecific polygenic genetic risk scores (Paquette et al., 2017; Futema et al., 2018; Rader and Sheth, 2019; Sharifi et al., 2019). Gene panels currently dominate FH molecular diagnostics and can be either customized on demand by individual labs or offered as designed commercial kits (Johansen et al., 2014).

Of course, there are some limitations of NGS technology. Middle-range insertions and deletions containing reads were unable to be mapped with earlier versions of alignment software, while recent updates have overcome those issues by extending the range of mapping quality values. The initial limit was dramatically increased to approximately 90 bp. The next limitation is the existence of pseudogenes. Due to high genomic sequence homology with genes of interest, it is very difficult or impossible to distinguish between reads originating from genes and their pseudogenes. Other limitations also exist, but these two are the most important.

Another methodological issue is that analysis of large intragenic rearrangements, which can be causal in many cases. These events can account for between 2 and 20% of all positive cases in some populations (Marduel et al., 2010; Pirillo et al., 2017; Tichý et al., 2017; Sun et al., 2018). Although multiplex ligation-dependent probe amplification is still the gold standard for the identification of large intragenic rearrangements, the analysis of copy number variations (CNVs) from NGS data can also achieve very good results (Marmontel et al., 2018) and has been gradually introduced into standard diagnostic schemes in routine labs. The opportunity to obtain information about the genomic sequence of many genes and about the potential presence of rearrangements in these genes at the same time in one step is the main advantage of the NGS approach.

Another strategy to detect CNVs is array comparative genomic hybridization (aCGH) (Ahmad and Iqbal, 2012), which evaluates patterns of intensities on single nucleotide polymorphism (SNP) microarrays to detect differences in allele dosages over a wide stretch of DNA. However, this method requires complex infrastructure and analytical tools for SNP microarrays. aCGH has relatively limited use in germinal genetics, and due to the special equipment needed, it is not common in standard diagnostic labs.

### Frequent Genetic Causes of FH

Most cases of FH are caused by defects in the gene for LDL receptor (*LDLR*) or for apolipoprotein B-100 (*APOB*) and rarely by variants in the gene for PCSK9 that lead to its overproduction (gain-of-function variants). Other variants in genes causing the FH phenotype have been described sporadically: for example, gene variations at *STAP1* or *APOE* loci (Santos et al., 2017). The phenotype of homozygous FH can also be caused by variants in the *LDLRAP1* gene, which are associated with an autosomal recessive form of the disease. Only a partial correlation between the genotype and phenotype has been reported in FH subjects. Higher LDL-C and a more severe phenotype are associated with so-called “null variants” in the *LDLR* gene, which lead to a decrease in LDL receptor activity below 2% of normal, while in the so-called “defective variants”, LDL receptor activity remains between 2 and 70% of normal function.

As mentioned above, pathogenic variants in the *LDLR* gene are the most common cause of FH. In most populations there is a very wide spectrum of LDLR variants. As we have published previously (Tichý et al., 2012), only 3 most common Czech pathogenic *LDLR* variants (Table 1) are relatively common in neighboring countries as well. Similar situation can be documented when reviewing the available literature worldwide (Bertolini et al., 2013; Komarova et al., 2013; Bañares et al., 2017; Alhababi and Zayed, 2018; Pek et al., 2018; Sun et al., 2018).

**TABLE 1:** The most frequent pathogenic variants in the *LDLR* gene and their frequencies detected in Czech Republic.

Variant at cDNA level	Variant at protein level	Type of variant	Location	Frequency (%)
c.1775G > A	p.(Gly592Glu)	Missense	Exon 12	16.77
c.798T > A	p.(Asp266Glu)	Missense	Exon 5	14.29
c.1061A > C	p.(Asp354Ala)	Missense	Exon 8	3.90
c.626G > A	p.(Cys209Tyr)	Missense	Exon 4	3.55
c.1246C > T	p.(Arg416Trp)	Missense	Exon 9	2.84
c.67 + 3968_940	Exon2_6dup	Gross		2.48
+ 296dup		duplication		
c.1186 + 700_2141	Exon9_14del	Gross deletion		2.13
−545del				
c.1567G > A	p.(Val523Met)	Missense	Exon 10	1.60
c.662A > G	p.(Asp221Gly)	Missense	Exon 4	1.51
c.1474G > A	p.(Asp492Asn)	Missense	Exon 10	1.51

*Frequency is calculated as a percentage of the particular variant of all pathogenic variants in the LDLR gene found in the Czech population.*

Ten *LDLR* gene variants presented in Table 1 account for approximately 50% of all pathogenic variants in the Czech Republic. Except for the variant p.(Arg3527Gln) in the *APOB* gene, no other pathogenic variants within the less frequent FH-causing genes have been shown to be prevalent in this population. Some of the common causal variants in these genes are presented in Table 2.

**TABLE 2:** Frequent pathogenic gene variants responsible for the development of FH (apart from the *LDLR* gene).

Gene	OMIM	Most frequent genetic mutations
APOB	107730	p.(Arg3527Gln) (Fernández-Higuero et al., 2015); p.(Arg3527Trp) (Yang et al., 2007)
PCSK9	607786	p.(Ser127Arg) (Abifadel et al., 2003); p.(Asp374Tyr) (Timms et al., 2004; Leren, 2004, Naoumova et al., 2005)
LDLRAP1	605747	p.(Ala145Serfs*26) (Arca et al., 2002); p.(Trp22*) (Garcia et al., 2001)
LIPA	613497	p.(Leu200Pro) (Anderson et al., 1994); p.(Gly266*) (Aslanidis et al., 1996)
ABCG5/ABCG8	605459/605460	ABCG8 p.(Trp361*) (Berge et al., 2000); ABCG5 p.(Arg408*) (Lee et al., 2001)

Identification of a causal variant in one of the genes responsible for the development of FH confirms the diagnosis of FH and thus a lifelong elevation of LDL-C. Most importantly, identification of the variant is crucial for successful cascade screening, which enables unambiguous confirmation or exclusion of FH in the proband’s family members. Moreover, identification of the variant in the family increases compliance of family members to undergo the examination, which is supported by findings from the Czech national database. In families with a known causal variant, the number of FH patients per family is on average 1.77, while in families without this information, it is only 1.18 (Vrablik et al., 2018). In the Czech Republic, we have found 226 different pathogenic variants—the p.(Arg3527Gln) variant in the *APOB* gene, 2 variants in the *PCSK9* gene and 223 variants in the *LDLR* gene. More information regarding the genetic analysis of the Czech MedPed cohort can be found in the articles by Tichý et al. (2012, 2017).

Recent community studies from the United States underline the benefits of genetic diagnostics, as these have shown that identification of a variant is an independent predictor of a manifest atherosclerotic cardiovascular disease in hypercholesterolemic patients (Khera et al., 2016). It is important to note that a negative result in the genetic examination does not completely rule out the possibility of FH. Such a result can be caused by low sensitivity of the methods used, the position of the variant being outside the analyzed part of the gene or a defect located in another gene. Additionally, some patients with a clinical diagnosis of FH can have polygenic hypercholesterolemia, as discussed below.

Recently, the diagnostic possibilities have improved significantly with the use of NGS. In a recent study using a whole-exome sequencing approach, the causal variant was discovered in 20% of patients with a definite clinical diagnosis of FH in whom previous DNA analysis found no variant (Futema et al., 2014). On the other hand, targeted NGS in a cohort of hypercholesterolemic patients in a primary care setting revealed an FH-causing variant in only 2% of the individuals examined (Norsworthy et al., 2014). Thus, appropriate selection of patients for genetic analysis remains a crucial step in FH identification.

### Occasional Variants in “Non-traditional Genes”

Typically, novel, rare FH-associated variants have been discovered through pedigree studies. This was the case for the identification of *CYP27A1* (cytochrome P450, subfamily XXVIIA, polypeptide 1), *LIPA* (lysosomal lipase A), *LIPC* (hepatic lipase), *LIPG* (endothelial lipase), *CYP7A1* (cytochrome P450 family 7 subfamily A member 1), *PNPLA5* (patatin-like phospholipase domain containing 5) and some other gene variants responsible for the FH phenotype in particular families (Lange et al., 2014; Al-Allaf et al., 2015; Pirillo et al., 2017; Corral et al., 2018; Mikhailova et al., 2019; Table 3).

**TABLE 3:** Overview of genetic causes of the FH phenotype.

Gene	Protein	Protein function	Mode of inheritance	Resulting effect
LDLR	Low-density lipoprotein receptor	Cell surface receptor that plays an important role in cholesterol homeostasis, transporting cholesterol through cell surface	AD	↓ binding affinity of LDL-receptor protein
APOB	Apolipoprotein B	Major protein of LDL particle, ligand for LDL receptor	AD	↓ binding affinity of APOB to LDL-receptor
PCSK9	Proprotein-convertase subtilisin kexin 9	Protease that plays a role in LDL-receptor degradation	AD	↑ LDL-receptor protein degradation
STAP1	Signal-transducing adaptor protein-1	Unknown	AD	Unknown/incomplete association with hypercholesterolemia
LDLRAP1	Low-density lipoprotein receptor adaptor protein 1	Adaptor protein that interacts with cytoplasmatic tail of LDL receptor, promoting LDL particle internalization	AR	LDL-receptor dysfunction
LIPA	Lysosomal acid lipase alias cholesterol esters lipase	Hydrolysis of cholesterol esters or triglycerides	AR	APOB overproduction, upregulation of HMGCoA reductase
ABCG5/ABCG8	Sterolin-1 and 2	Transporters required for secretion of cholesterol into bile	AR	↑ absorption of plant sterols
PNPLA5	Patatin-like phospholipase domain-containing protein 5	Influencing adipocyte differentiation, triglyceride hydrolysis	AR	Possibly lipolysis impairment

*AR, autosomal recessive; AD, autosomal dominant; HMGCoA, hydroxy-methyl glutaryl coenzyme A.*

### “False” Familial Hypercholesterolemia Cases

The seemingly simple picture of FH is further complicated by the fact that not all cases described as FH are correctly classified and that they do not fulfill the universally recognized criteria of the disease. Sometimes, these situations are reported as FH phenocopies (Page et al., 2020).

A typical example of such a misclassification is represented by variants of the *ABCG5/G8* transporter genes. In fact, these variants cause sitosterolaemia (Berge et al., 2000; Hubacek et al., 2001), a disease with an autosomal recessive mode of inheritance, where high plasma levels of cholesterol in fact represent a severe elevation of plant sterol plasma concentration. Common enzymatic assays do not discriminate between cholesterol and plant sterols; thus, sitosterolaemia is frequently confused with FH (Moghadasian et al., 2002). Although sitosterolaemia was originally thought to occur in the general population with a frequency of approximately 1:1,000,000, a recent study (Brinton et al., 2018) suggested a prevalence of approximately 1:2,000. This finding is of utmost importance, as 5–10% of patients with the FH phenotype may actually be affected by sitosterolaemia. “Reclassification” of these FH cases accordingly would have important therapeutic implications as the therapy of choice of the latter condition is a cholesterol absorption blocker (ezetimibe) and not a statin.

Similarly, variants of the LDL receptor adaptor protein 1 gene [*LDLRAP1*, originally named the ARH (autosomal recessive hypercholesterolemia) gene] (Garcia et al., 2001) should formally not be considered FH-causing, as the mode of inheritance is recessive. However, this condition cannot be clinically distinguished from the homozygous form of autosomal dominant FH, as its consequences are similar to those of the “classical” form of the disease.

Finally, severe FH-like hypercholesterolemia might infrequently be mimicked by very high plasma levels of lipoprotein (a) [Lp(a)] (Zlatohlavek et al., 2008; Langsted et al., 2016). When LDL-C concentration is measured, it always comprises the amount of cholesterol carried within Lp(a) particles. Such a situation would be connected to statin resistance, as Lp(a) levels cannot be reduced with common lipid-lowering therapy. Moreover, high Lp(a) concentrations in the context of elevated LDL-C represent an additional factor aggravating atherothrombotic risk. Thus, all patients with the FH phenotype must be screened for Lp(a) levels as well (Langsted et al., 2016).

### Polygenic Familial Hypercholesterolemia (Pseudo-FH)

The term “polygenic familial hypercholesterolemia” was probably first mentioned by Talmud et al. (2013), although simultaneous analyses of more genes to describe the polygenic nature of hypercholesterolemia are much older (for example, Pedersen and Berg, 1990; Poledne et al., 1994).

The last decade has witnessed an intensive scientific debate triggered by the fact that the majority of subjects with probable or definite FH based on clinical and biochemical criteria cannot be confirmed genetically (e.g., do not have any detectable variation in *LDL-R*, *APOB*, and *PCSK9*). Efforts to identify novel causal genes using the results of genome-wide association studies have been typically unsuccessful at the population level, albeit some rare cases of FH caused by rare variants in different genes have been described (see information above).

Many potential candidate genes, however, have failed to be confirmed as FH-causing genes. For example, sortilin (*SORT-1*) has been documented to play an important role in LDL particle internalization, and common variants of this gene represent an important and highly significant determinant of plasma cholesterol values at the population level. Nevertheless, no *SORT-1* variants have been detected in almost 900 FH patients negatively tested for variants at *LDL-R*, *APOB*, or *PCSK9* loci (Tveten et al., 2012).

It is hypothesized that (at least some) FH variant-negative patients are in fact carriers of a high number of commonly present genetic variants associated with increased plasma cholesterol values, thus having polygenic FH.

Variant negative FH cases are sometimes referred to as “pseudo-FH” or “polygenic FH” patients.

In 2013, Talmud and co-workers proposed 12 common single nucleotide variants of the *APOE*, *SORT1*, *LDLR*, *APOB, PCSK9, HFE, ABCG8, NYNRIN*, *MYLIP*, *SLC-22* and *ST3GAL4* genes identified through the Global Lipids Genetics Consortium (GLGC; definition at http://lipidgenetics.org), which could be useful for the detection of pseudo-FH subjects. The authors concluded that FH-causing variant-negative “pseudo-FH” patients have a significantly higher mean weighted LDL-C genetic score than the general population.

Later, the 12-SNP gene score was reduced to a 6-SNP gene score (Futema et al., 2015). This 6-SNP LDL-C score has been found to be increased in variant-negative FH patients of Israeli origin with respect to controls from the general population. This further confirms that a significant accumulation of common gene variants of small effect can lead to severe hypercholesterolemia that might not be distinguished from autosomal dominant FH (Durst et al., 2017).

All the abovementioned models suppose simple additive effects of genetic and environmental effects. However, due to gene-gene or gene-environment interactions, the final cholesterol values can be much higher (however, also much lower) than expected from these models (Ritchie, 2015). Currently, this is primarily a hypothesis, but there are examples of gene-environment (Hubacek et al., 2003; Shirts et al., 2012; Kim et al., 2013) and gene-gene interactions (Hubacek et al., 2008; Ma et al., 2012; Grave et al., 2016) modifying plasma cholesterol levels at the general population level; thus, there is no reason that such effects would not work for FH patients. To date, no results have been published for FH patient populations, although some studies (Gaspar and Gaspar, 2019) focused on variable FH penetration indirectly support this model.

### Additional Common Gene Variants as the Basis for Novel Polygenic FH Scores

It could be speculated that the gene score can also comprise SNPs occurring within the genes known to cause monogenic forms of FH. Such an approach is plausible, as the products of these genes determine important pathways of cholesterol absorption and metabolism. The putative genes that could be included in this extended gene score include, for example, the genes for BRAP (BRCA-1 associated protein), CETP (cholesterol ester transfer protein), FADS (fatty acid desaturase) and PPP1R3B (protein phosphatase 1, regulatory subunit 3B). Interestingly, they were suggested by the same authors who presented the reduced 6-SNP-based score of polygenic FH (Futema et al., 2015).

The accumulation of common cholesterol-increasing alleles could lead to a condition mimicking and/or worsening a coexisting monogenic form of FH. On the other hand, the possibility of “camouflaging” the FH phenotype by the accumulation of common alleles associated with lower plasma cholesterol levels can occur in the FH population as well, despite there being no literature on the topic so far.

## Ethnicity/Population-Specific SNPs

One could speculate about the utility of a single universal gene score, especially as during the last decade we have witnessed increased interest in the implementation of the principles of personalized medicine (Currie and Delles, 2018). The impact of GWAS-detected, lipid trait-associated SNPs could significantly differ in different populations (Hubacek et al., 2017, 2019). For example, in the Japanese population (Tada et al., 2018), variants within the *ABO* gene have been identified as significantly modifying plasma lipoprotein metabolism, while “the usual suspects” (genes for *SORT-1*, *LDLR* and *HMGCR*) showed rather negligible effects. Importantly, identical variants could have different effects on plasma lipids and the frequencies of genetic variants can differ between different ethnicities (Han et al., 2019); thus, a different gene score should be introduced for populations of different ethnic backgrounds.

Given that hundreds of SNPs are probably significantly associated with plasma cholesterol levels, we assume that the gene score needs to be more complex and probably includes dozens of individual SNPs.

## Treatment Considerations

The treatment of FH patients must be comprehensive and always include non-pharmacological approaches (promoted from childhood—in this case with greater success than later age) and (combination) pharmacotherapy. Patients with FH in childhood and adolescence, as well as women of childbearing potential and women during pregnancy, require a special approach to treatment. A healthy diet and physical activity alone in patients with FH never lead to sufficient changes in the lipoprotein profile, and pharmacotherapy remains essential. Nevertheless, a healthy diet with adequate (as high as possible) physical activity has a positive effect on all known risk factors for atherosclerosis and, most likely, those that have not yet been identified. Pharmacotherapy for patients with FH is based on highly effective statins with a long half-life allowing administration at any time of the day and thus favorably affecting patient adherence. We can titrate the treatment to the maximum dose or at least to a high intensity (atorvastatin 40–80 mg, rosuvastatin 20-40 mg), which is a procedure necessary in patients with partial statin intolerance (Vrablik et al., 2014). Monotherapy with a high-intensity statin usually reduces LDL-C by 50%. Once the maximum tolerated dose of a statin is not sufficient to reach the LDL-cholesterol goal, a combination of statin + ezetimibe should be introduced into the treatment of FH in the next step. Ezetimibe can also be added to combinations for patients who cannot tolerate high doses of statins. Given the relatively lower efficacy of ezetimibe monotherapy (due to the compensatory increase in endogenous cholesterol production in the liver with cholesterol absorption blockage), we always try to guide patients to at least a small dose of statins (e.g., 5 mg atorvastatin or rosuvastatin daily or an alternative dosing). The addition of ezetimibe to a statin reduces LDL-C levels by an additional 20–25%. The use of resins (bile acid sequestrants) is limited mostly due to their poor tolerance; they are used mainly in pediatric patients with FH. On the other hand, the population of patients with FH represents a target group in which we continue to use resins as part of a comprehensive effort to maximize the LDL-C reducing effect. To date, the latest additions to the family of lipid-lowering drugs are monoclonal antibodies against PCSK9. PCSK9 is a protein involved in both intracellular and extracellular regulation of LDL cholesterol receptor expression. One of the functions of PCSK9 is the formation of a complex of PCSK9 with the LDL receptor and its internalization in the endosome. Binding of PCSK9 to the LDL receptor in the cell prevents the normal course of receptor recycling and re-exposure to the plasma membrane. Instead, the LDL receptor-PCSK9 complex is transferred intracellularly to the lysosome, where it undergoes degradation. The number of LDL receptors on the cell surface is thus reduced depending on the presence of PCSK9. Anti-PCSK9 antibodies are capable of increasing LDL receptor expression and ultimately lowering LDL-C levels by up to twenty percent (Ogura, 2018). Two agents, alirocumab and evolocumab, have been introduced into clinical practice.

Lipoprotein apheresis should be considered a therapeutic option for patients with severe hypercholesterolemia who have persistently elevated LDL-C levels despite optimized and intensive drug therapy (Bambauer et al., 2012). It is an extracorporeal elimination technique that removes LDL particles but usually also some other atherogenic lipoproteins, such as Lp(a) or triglyceride-rich lipoproteins, from the circulation. The main indications for lipoprotein apheresis are a diagnosis of homozygous FH, severe heterozygous FH poorly responding to standard therapy, and patients with Lp(a) increase resistant to pharmacotherapy (Blaha et al., 2017a). Lipoprotein apheresis is also a potent therapeutic player that impacts inflammation and related mediators. A large body of evidence on this is available (Blaha et al., 2017b; Stefanutti and Zenti, 2018).

## Future Directions

FH variants leading to very high plasma cholesterol levels are not necessarily associated with premature atherosclerosis and mortality (Williams et al., 1986). Interestingly, even Brown and Goldstein (1983) in their pioneering works mentioned the lack of association between plasma cholesterol values in FH patients and the prognosis of the disease.

Development of genetic testing has enabled better discrimination between “classical” FH and other forms of hypercholesterolemia, as well as improvement in our understanding of the pathophysiology of the disease. As elegantly summarized in a consensus statement published by Sturm et al. (2018), genetic testing in FH:

– provides a definitive molecular diagnosis of FH
– provides prognostic and risk stratification information and improves outcomes
– facilitates family-based cascade testing
– allows for precision during genetic counseling
– has implications for therapeutic choices in FH
– has value to the pediatric FH patient population.

However, with developments in the genetic diagnosis and the availability of high-throughput technologies, many “innocent” genetic variants have been identified in the genes recognized as causally linked to FH. Thus, based on the consensus of the American College of Medical Genetics (ACMG), the ClinVar initiative has been established to determine the likely pathogenicity of variants in *LDLR/APOB/PCSK9* genes reported in patients with clinical FH (Richards et al., 2015; Iacocca et al., 2018). Classifications include “definitely not”, “likely not pathogenic,” “variants of unknown significance” (VUS), “likely pathogenic” and “definitely pathogenic.” While more than 70% of the 2314 published *LDLR* variants are classified as “likely” or “definitely pathogenic,” only 10% of the *APOB* and 13% of *PCSK9* variants are classified as such (Iacocca et al., 2018).

The next primary goal in the management of FH is to increase medical community awareness of FH and the active search for patients; this should lead to an increase in the number of diagnosed and well-managed patients and, even more importantly, a substantial increase in the number of examined members of affected families. Many initiatives focusing on FH detection have been launched recently. In Australia, Asia-Pacific countries and South America, the “Ten Countries Study” was successfully conducted by Watts et al. (2016). Another rapidly developing FH project creating a platform for mutual interaction of FH patients and health care professionals, “The FH Foundation”, has been developing since 2011 in the United States (O’Brien et al., 2014). In Europe, the “FH Studies Collaboration” project led by K. Ray and supported by the European Atherosclerosis Society (EAS) has evolved into a multinational project aimed at providing consolidated data on FH worldwide together with the creation of a universal database platform for data collection (Vallejo-Vaz et al., 2015). The ScreenProFH project, endorsed by the International Atherosclerosis Society and embedded in the FHSC initiative, helps to enhance FH screening activities in the Central, Eastern and Southern European region as well as Central Asia and is described in detail in a separate article (Ceska et al., 2017). The Czech MedPed project is actively participating in and/or collaborating with all these international activities. Undoubtedly, these coordinated international efforts should increase the chances of achieving the principal goal—to identify, diagnose and provide treatment for all FH patients early enough to prevent the development of atherosclerotic vascular complications and avoid unnecessary premature death.

## Author Contributions

MV and JAH contributed substantially to the concept. LT, MS, MV, JAH, TF, and VB were involved in interpretation of the data. All authors were involved in drafting of the manuscript, provided critical revisions for important intellectual content, approved the final version submitted for publication, and agreed to be accountable for all aspects of the work.

## Conflict of Interest

The authors declare that the research was conducted in the absence of any commercial or financial relationships that could be construed as a potential conflict of interest.

## Footnotes

1 https://www.who.int/news-room/fact-sheets/detail/the-top-10-causes-of-death

## References

[B1] Abifadel M. Varret M. Rabès J. P. Allard D. Ouguerram K. Devillers M. (2003). Mutations in PCSK9 cause autosomal dominant hypercholesterolemia. *Nat. Genet*. 34 154–156. 10.1038/ng1161 12730697

[B2] Ahmad A. Iqbal M. A. (2012). Significance of genome-wide analysis of copy number alterations and UPD in myelodysplastic syndromes using combined CGH - SNP arrays. *Curr. Med. Chem.* 19 3739–3747. 10.2174/092986712801661121 22680919

[B3] Al-Allaf F. A. Athar M. Abduljaleel Z. Taher M. M. Khan W. Ba-Hammam F. A. (2015). Next generation sequencing to identify novel genetic variants causative of autosomal dominant familial hypercholesterolemia associated with increased risk of coronary heart disease. *Gene* 565 76–84. 10.1016/j.gene.2015.03.064 25839937

[B4] Alhababi D. Zayed H. (2018). Spectrum of mutations of familial hypercholesterolemia in the 22 Arab countries. *Atherosclerosis* 279 62–72. 10.1016/j.atherosclerosis.2018.10.022 30415195

[B5] Anderson R. A. Byrum R. S. Coates P. M. Sando G. N. (1994). Mutations at the lysosomal acid cholesteryl ester hydrolase gene locus in Wolman disease. *Proc. Natl. Acad. Sci. U.S.A*. 91 2718–2722. 10.1073/pnas.91.7.2718 8146180 PMC43441

[B6] Arca M. Zuliani G. Wilund K. Campagna F. Fellin R. Bertolini S. (2002). Autosomal recessive hypercholesterolaemia in Sardinia, Italy, and mutations in ARH: a clinical and molecular genetic analysis. *Lancet*. 359 841–847. 10.1016/S0140-6736(02)07955-2 11897284

[B7] Aslanidis C. Ries S. Fehringer P. Büchler C. Klima H. Schmitz G. (1996). Genetic and biochemical evidence that CESD and Wolman disease are distinguished by residual lysosomal acid lipase activity. *Genomics* 33 85–93. 10.1006/geno.1996.0162 8617513

[B8] Bambauer R. Bambauer C. Lehmann B. Latza R. Schiel R. (2012). LDL-apheresis: technical and clinical aspects. *Sci. World J.* 2012:314283. 10.1100/2012/314283 PMC3361163 22654591

[B9] Bañares V. G. Corral P. Medeiros A. M. Araujo M. B. Lozada A. Bustamante J. (2017). Preliminary spectrum of genetic variants in familial hypercholesterolemia in Argentina. *J. Clin. Lipidol*. 11 524–531. 10.1016/j.jacl.2017.02.007 28502510

[B10] Benn M. Watts G. F. Tybjaerg-Hansen A. Nordestgaard B. G. (2012). Familial hypercholesterolemia in the danish general population: prevalence, coronary artery disease, and cholesterol-lowering medication. *J. Clin. Endocrinol. Metab.* 97 3956–3964. 10.1210/jc.2012-1563 22893714

[B11] Berge K. E. Tian H. Graf G. A. Yu L. Grishin N. V. Schultz J. (2000). Accumulation of dietary cholesterol in sitosterolemia caused by mutations in adjacent ABC transporters. *Science* 290 1771–1775. 10.1126/science.290.5497.1771 11099417

[B12] Bertolini S. Pisciotta L. Rabacchi C. Cefalù A. B. Noto D. Fasano T. (2013). Spectrum of mutations and phenotypic expression in patients with autosomal dominant hypercholesterolemia identified in Italy. *Atherosclerosis* 227 342–348. 10.1016/j.atherosclerosis.2013.01.007 23375686

[B13] Blaha V. Blaha M. Lanska M. Solichova D. Kujovska Krcmova L. Havel E. (2017a). Lipoprotein apheresis in the treatment of dyslipidaemia - the Czech Republic experience. *Physiol. Res*. 66 S91–S100. 28379034 10.33549/physiolres.933584

[B14] Blaha V. Blaha M. Solichova D. Kujovska Krcmova M. Lanska M. Havel E. (2017b). Antioxidant defense system in familial hypercholesterolemia and the effects of lipoprotein apheresis. *Atheroscl. Suppl.* 30 159–165. 10.1016/j.atherosclerosissup.2017.05.002 29096832

[B15] Brinton E. A. Hopkins P. N. Hegele R. A. Geller A. S. Polisecki E. Y. Diffenderfer M. R. (2018). The association between hypercholesterolemia and sitosterolemia, and report of a sitosterolemia kindred. *J. Clin. Lipidol.* 12 152–161. 10.1016/j.jacl.2017.10.013 29169939

[B16] Brown M. S. Goldstein J. L. (1983). Lipoprotein metabolism in the macrophage: implications in cholesterol deposition in atherosclerosis0. *Annu. Rev. Biochem.* 52 223–261. 6311077 10.1146/annurev.bi.52.070183.001255

[B17] Ceska R. Freiberger T. Vaclova M. Aleksicova T. Votavova L. Vrablik M. (2017). ScreenPro FH: from the Czech MedPed to international collaboration. ScreenPro FH is a participating project of the EAS-FHCS. *Physiol Res* 66(Suppl. 1), S85–S90. 10.33549/physiolres.933599 28379033

[B18] Corral P. Geller A. S. Polisecki E. Y. Lopez G. I. Bañares V. G. Cacciagiu L. (2018). Unusual genetic variants associated with hypercholesterolemia in Argentina. *Atherosclerosis* 277 256–261. 10.1016/j.atherosclerosis.2018.06.009 30270055

[B19] Cuchel M. Bruckert E. Ginsberg H. N. Raal F. J. Santos R. D. Hegele R. A. (2014). Homozygous familial hypercholesterolaemia: new insights and guidance for clinicians to improve detection and clinical management. A position paper from the consensus panel on familial hypercholesterolaemia of the european atherosclerosis society. *Eur. Heart J*. 35 2146–2157. 10.1093/eurheartj/ehu274 25053660 PMC4139706

[B20] Currie G. Delles C. (2018). Precision medicine and personalized medicine in cardiovascular disease. *Adv. Exp. Med. Biol.* 1065 589-605. 10.1007/978-3-319-77932-4_36 30051409

[B21] Durst R. Ibe U. K. Shpitzen S. Schurr D. Eliav O. Futema M. (2017). Molecular genetics of familial hypercholesterolemia in Israel-revisited. *Atherosclerosis* 257 55–63. 10.1016/j.atherosclerosis.2016.12.021 28104544

[B22] Farhan S. M. Hegele R. A. (2014). Exome sequencing: new insights into lipoprotein disorders. *Curr. Cardiol. Rep.* 16:507. 10.1007/s11886-014-0507-2 24893940

[B23] Fernández-Higuero J. A. Etxebarria A. Benito-Vicente A. Alves A. C. Arrondo J. L. Ostolaza H. (2015). Structural analysis of APOB variants, p.(Arg3527Gln), p.(Arg1164Thr) and p.(Gln4494del), causing Familial Hypercholesterolaemia provides novel insights into variant pathogenicity. *Sci. Rep.* 5:18184. 10.1038/srep18184 26643808 PMC4672294

[B24] Futema M. Bourbon M. Williams M. Humphries S. E. (2018). Clinical utility of the polygenic LDL-C SNP score in familial hypercholesterolemia. *Atherosclerosis* 277 457–463. 10.1016/j.atherosclerosis.2018.06.006 30270085

[B25] Futema M. Plagnol V. Li K. Whittall R. A. Neil H. A. Seed M. (2014). Whole exome sequencing of familial hypercholesterolaemia patients negative for LDLR/APOB/PCSK9 mutations. *J. Med. Genet*. 51 537–544. 10.1136/jmedgenet-2014-102405 24987033 PMC4112429

[B26] Futema M. Shah S. Cooper J. A. Li K. Whittall R. A. Sharifi M. (2015). Refinement of variant selection for the LDL cholesterol genetic risk score in the diagnosis of the polygenic form of clinical familial hypercholesterolemia and replication in samples from 6 countries. *Clin. Chem.* 61 231–238. 10.1373/clinchem.2014.231365 25414277 PMC4892342

[B27] Garcia C. K. Wiklund K. Arca M. Zuliani G. Fellin R. Maioli M. (2001). Autosomal recessive hypercholesterolemia caused by mutations in a putative LDL receptor adaptor protein. *Science* 292 1394–1398. 10.1126/science.1060458 11326085

[B28] Gaspar I. M. Gaspar A. (2019). Variable expression and penetrance in Portuguese families with Familial Hypercholesterolemia with mild phenotype. *Atheroscl. Suppl*. 36 28–30. 10.1016/j.atherosclerosissup.2019.01.006 30876530

[B29] Goldstein J. L. Kottke B. A. Brown M. S. (1982). Biochemical genetics of LDL receptor mutations in familial hypercholesterolemia. *Prog. Clin. Biol. Res.* 103(Pt. B), 161–176. 6298809

[B30] Grave N. Tovo-Rodrigues L. da Silveira J. Rovaris D. L. Dal Bosco S. M. Contini V. (2016). A vitamin D pathway gene-gene interaction affects low-density lipoprotein cholesterol levels. *J. Nutr. Biochem.* 38 12–17. 10.1016/j.jnutbio.2016.08.002 27721113

[B31] Guirgis F. W. Donnelly J. P. Dodani S. Howard G. Safford M. M. Levitan E. B. (2016). Cholesterol levels and long-term rates of community-acquired sepsis. *Crit Care*. 20:408. 10.1186/s13054-016-1579-8 28010729 PMC5180408

[B32] Han S. Hwang M. Y. Yoon K. Kim Y. K. Kim Y. J. Kim B. J. (2019). Exome chip-driven association study of lipidemia in >14,000 Koreans and evaluation of genetic effect on identified variants between different ethnic groups. *Genet. Epidemiol*. 43 617–628. 10.1002/gepi.22208 31087446

[B33] Hegele R. A. Ban M. R. Cao H. McIntyre A. D. Robinson J. F. Wang J. (2015). Targeted next-generation sequencing in monogenic dyslipidemias. *Curr. Opin. Lipidol.* 26 103–113. 10.1097/MOL.0000000000000163 25692347

[B34] Hu P. Dharmayat K. I. Stevens C. A. T. Sharabiani M. T. A. Jones R. S. Watts G. F. (2020). Prevalence of Familial Hypercholesterolemia Among the general population and patients with atherosclerotic cardiovascular disease: a systematic review and meta-analysis. *Circulation* 141 1742–1759. 10.1161/CIRCULATIONAHA.119.044795 32468833

[B35] Hubacek J. A. (2009). Eat less and exercise more - is it really enough to knock down the obesity pandemia? *Physiol. Res.* 58(Suppl. 1), S1–S6. 10.33549/physiolres.931855 19857030

[B36] Hubacek J. A. Adamkova V. Lanska V. Dlouha D. (2017). Polygenic hypercholesterolemia: examples of GWAS results and their replication in the Czech-Slavonic population. *Physiol. Res.* 66(Suppl. 1), S101–S111. 10.33549/physiolres.933580 28379035

[B37] Hubacek J. A. Berge K. E. Cohen J. C. Hobbs H. H. (2001). Mutations in ATP-cassette binding proteins G5 (ABCG5) and G8 (ABCG8) causing sitosterolemia. *Hum. Mutat.* 18 359–360. 10.1002/humu.1206 11668628

[B38] Hubacek J. A. Dlouha D. Adamkova V. Schwarzova L. Lanska V. Ceska R. (2019). The gene score for predicting hypertriglyceridemia: new insights from a Czech case-control study. *Mol. Diagn. Ther.* 23 555–562. 31222479 10.1007/s40291-019-00412-2

[B39] Hubacek J. A. Lanska V. Skodova Z. Adamkova V. Poledne R. (2008). Sex-specific interaction between APOE and APOA5 variants and determination of plasma lipid levels. *Eur. J. Hum. Genet.* 16 135–138. 10.1038/sj.ejhg.5201941 17957227

[B40] Hubacek J. A. Pitha J. Skodova Z. Poledne R. Lanska V. Waterworth D. M. (2003). Polymorphisms in CYP-7A1, not APOE, influence the change in plasma lipids in response to population dietary change in an 8 year follow-up; results from the Czech MONICA study. *Clin. Biochem.* 36 263–267. 10.1016/s0009-9120(03)00025-0 12810154

[B41] Iacocca M. A. Chora J. R. Carrie A. Freiberger T. Leigh S. E. Defesche J. C. (2018). ClinVar database of global familial hypercholesterolemia-associated DNA variants. *Hum. Mutat*. 39 1631–1640. 30311388 10.1002/humu.23634 PMC6206854

[B42] Johansen C. T. Dubé J. B. Loyzer M. N. MacDonald A. Carter D. E. McIntyre A. D. (2014). LipidSeq: a next-generation clinical resequencing panel for monogenic dyslipidemias. *J. Lipid. Res.* 55 765–772. 10.1194/jlr.D045963 24503134 PMC3966710

[B43] Khera A. V. Won H. H. Peloso G. M. Lawson K. S. Bartz T. M. Deng X. (2016). Diagnostic yield and clinical utility of sequencing familial hypercholesterolemia genes in patients with severe hypercholesterolemia. *J. Am. Coll. Cardiol.* 67 2578–2589. 10.1016/j.jacc.2016.03.520 27050191 PMC5405769

[B44] Kim D. S. Burt A. A. Ranchalis J. E. Jarvik E. R. Rosenthal E. A. Hatsukami T. S. (2013). Novel gene-by-environment interactions: APOB and NPC1L1 variants affect the relationship between dietary and total plasma cholesterol. *J. Lipid. Res.* 54 1512–1520. 10.1194/jlr.P035238 23482652 PMC3622343

[B45] Komarova T. Y. Korneva V. A. Kuznetsova T. Y. Golovina A. S. Vasilyev V. B. Mandelshtam M. Y. (2013). Familial hypercholesterolemia mutations in Petrozavodsk: no similarity to St. Petersburg mutation spectrum. *BMC Med. Genet.* 14:128. 10.1186/1471-2350-14-128 24373485 PMC3877960

[B46] Lange L. A. Hu Y. Zhang H. Xue C. Schmidt E. M. Tang Z. Z. (2014). Whole-exome sequencing identifies rare and low-frequency coding variants associated with LDL cholesterol. *Am. J. Hum. Genet*. 94 233–245. 10.1016/j.ajhg.2014.01.010 24507775 PMC3928660

[B47] Langsted A. Kamstrup P. R. Benn M. Tybjaerg-Hansen A. Nordestgaard B. G. (2016). High lipoprotein(a) as a possible cause of clinical familial hypercholesterolaemia: a prospective cohort study. *Lancet Diab. Endocrinol.* 4 577–587. 10.1016/S2213-8587(16)30042-0 27185354

[B48] Lee M. H. Lu K. Hazard S. Yu H. Shulenin S. Hidaka H. (2001). Identification of a gene, ABCG5, important in the regulation of dietary cholesterol absorption. *Nat. Genet*. 27 79–83. 10.1038/83799 11138003 PMC1350991

[B49] Leigh S. Futema M. Whittall R. Taylor-Beadling A. Williams M. denDunnen J. T. (2017). The UCL low-density lipoprotein receptor gene variant database: pathogenicity update. *J. Med. Genetics* 54 217–223. 10.1136/jmedgenet-2016-104054 27821657 PMC5502305

[B50] Leren T. P. (2004). Mutations in the PCSK9 gene in Norwegian subjects with autosomal dominant hypercholesterolemia. *Clin. Genet*. 65 419–422. 10.1111/j.0009-9163.2004.0238.x 15099351

[B51] Loscalso J. (2004). *Molecular Mechanisms of Atherosclerosis.* Boca Raton, FL: CRC press.

[B52] Ma L. Brautbar A. Boerwinkle E. Sing C. F. Clark A. G. Keinan A. (2012). Knowledge-driven analysis identifies a gene-gene interaction affecting high-density lipoprotein cholesterol levels in multi-ethnic populations. *PLoS Genet*. 8:e1002714. 10.1371/journal.pgen.1002714 22654671 PMC3359971

[B53] Marduel M. Carrié A. Sassolas A. Devillers M. Carreau V. Di Filippo M. (2010). Molecular spectrum of autosomal dominant hypercholesterolemia in France. *Hum. Mutat.* 31 E1811–E1824. 10.1002/humu.21348 20809525 PMC3152176

[B54] Marmontel O. Charrière S. Simonet T. Bonnet V. Dumont S. Mahl M. (2018). Single, short in-del, and copy number variations detection in monogenic dyslipidemia using a next-generation sequencing strategy. *Clin. Genet.* 94 132–140. 10.1111/cge.13250 29572815

[B55] Mikhailova S. Ivanoshchuk D. Timoshchenko O. Shakhtshneider E. (2019). Genes potentially associated with familial hypercholesterolemia. *Biomolecules* 9:807. 10.3390/biom9120807 31795497 PMC6995538

[B56] Moghadasian M. H. Frohlich J. J. Scudamore C. H. (2002). Specificity of the commonly used enzymatic assay for plasma cholesterol determination. *J. Clin. Pathol.* 55 859–861. 10.1136/jcp.55.11.859 12401826 PMC1769797

[B57] Naoumova R. P. Tosi I. Patel D. Neuwirth C. Horswell S. D. Marais A. D. (2005). Severe hypercholesterolemia in four British families with the D374Y mutation in the PCSK9 gene: long-term follow-up and treatment response. *Arterioscl. Thromb. Vasc. Biol*. 25 2654–2660. 10.1161/01.ATV.0000190668.94752.ab 16224054

[B58] Norsworthy P. J. Vandrovcova J. Thomas E. R. Campbell A. Kerr S. M. Biggs J. (2014). Targeted genetic testing for familial hypercholesterolaemia using next generation sequencing: a population-based study. *BMC Med. Genet.* 15:70. 10.1186/1471-2350-15-70 24956927 PMC4083361

[B59] O’Brien E. C. Roe M. T. Fraulo E. S. Peterson E. D. Ballantyne C. M. Genest J. (2014). Rationale and design of the familial hypercholesterolemia foundation CAscade SCreening for Awareness and DEtection of Familial Hypercholesterolemia registry. *Am. Heart J.* 167 342–349. 10.1016/j.ahj.2013.12.008 24576518

[B60] Ogura M. (2018). PCSK9 inhibition in the management of familial hypercholesterolemia. *J. Cardiol*. 71 1–7. 10.1016/j.jjcc.2017.07.002 28784313

[B61] Page M. M. Bell D. A. Watts G. F. (2020). Widening the spectrum of genetic testing in familial hypercholesterolaemia: will it translate into better patient, and population outcomes? *Clin. Genet.* 97 543–555. 31833051 10.1111/cge.13685

[B62] Paquette M. Chong M. Thériault S. Dufour R. Paré G. Baass A. (2017). Polygenic risk score predicts prevalence of cardiovascular disease in patients with familial hypercholesterolemia. *J. Clin. Lipidol.* 11 725.e5–732.e5. 10.1016/j.jacl.2017.03.019 28456682

[B63] Pedersen J. C. Berg K. (1990). gene interaction between the low-density lipoprotein receptor and apolipoprotein E loci affects lipid levels. *Clin. Genet.* 38 287–294. 10.1111/j.1399-0004.1990.tb03583.x 1980097

[B64] Pek S. L. T. Dissanayake S. Fong J. C. W. Lin M. X. Chan E. Z. L. Tang J. I. (2018). Spectrum of mutations in index patients with familial hypercholesterolemia in Singapore: single center study. *Atherosclerosis* 269 106–116. 10.1016/j.atherosclerosis.2017.12.028 29353225

[B65] Pirillo A. Garlaschelli K. Arca M. Averna M. Bertolini S. Calandra S. (2017). Spectrum of mutations in Italian patients with familial hypercholesterolemia: new results from the LIPIGEN study. *Atheroscl. Suppl.* 29 17–24. 10.1016/j.atherosclerosissup.2017.07.002 28965616

[B66] Poledne R. Hubacek J. Pisa Z. Pistulkova H. Valenta Z. (1994). Genetic markers in hypercholesterolemic and normocholesterolemic Czech children. *Clin. Genet.* 46 88–91. 10.1111/j.1399-0004.1994.tb04208.x 7988085

[B67] Rader D. J. Sheth S. (2019). Polygenic risk scores in familial hypercholesterolemia. *J. Am. Coll. Cardiol.* 74 523–525. 10.1016/j.jacc.2019.06.006 31345426

[B68] Richards S. Aziz N. Bale S. Bick D. Das S. Gastier-Foster J. (2015). Standards and guidelines for theinterpretation of sequence variants: a joint consensus recommendation of the american college of medical genetics and genomics and the association for molecular pathology. *Genet.Med.* 17 405–424. 25741868 10.1038/gim.2015.30 PMC4544753

[B69] Ritchie M. D. (2015). Finding the epistasis needles in the genome-wide haystack. *Methods Mol. Biol*. 1253 19–33. 10.1007/978-1-4939-2155-3_2 25403525

[B70] Santos R. D. Bourbon M. Alonso R. Cuevas A. Vasques-Cardenas N. A. Pereira A. C. (2017). Clinical and molecular aspects of familial hypercholesterolemia in Ibero-American countries. *J. Clin. Lipidol*. 11 160–166. 10.1016/j.jacl.2016.11.004 28391882

[B71] Sharifi M. Futema M. Nair D. Humphries S. E. (2019). Polygenic hypercholesterolemia and cardiovascular disease risk. *Curr. Cardiol. Rep.* 21:43. 10.1007/s11886-019-1130-z 31011892 PMC6477004

[B72] Shirts B. H. Howard M. T. Hasstedt S. J. Nanjee M. N. Knight S. Carlquist J. F. (2012). Vitamin D dependent effects of APOA5 polymorphisms on HDL cholesterol. *Atherosclerosis* 222 167–174. 10.1016/j.atherosclerosis.2012.02.030 22425169 PMC3334480

[B73] Sijbrands E. J. Westendorp R. G. Defesche J. C. de Meier P. H. Smelt A. H. Kastelein J. J. (2001). Mortality over two centuries in large pedigree with familial hypercholesterolaemia: family tree mortality study. *BMJ* 322 1019–1023. 10.1136/bmj.322.7293.1019 11325764 PMC31037

[B74] Stefanutti C. Zenti M. G. (2018). Lipoprotein apheresis and PCSK9-Inhibitors. impact on atherogenic lipoproteins and anti-inflammatory mediators in familial Hypercholesterolaemia. *Curr. Pharm. Des*. 24 3634–3637. 30360706 10.2174/1381612824666181025115658

[B75] Sturm A. C. Knowles J. W. Gidding S. S. Ahmad Z. S. Ahmed C. D. Ballantyne C. M. (2018). Clinical genetic testing for familial hypercholesterolemia: JACC scientific expert panel. *J. Am. Coll. Cardiol*. 72 662–680. 10.1016/j.jacc.2018.05.044 30071997

[B76] Sun D. Zhou B. Y. Li S. Sun N. L. Hua Q. Wu S. L. (2018). Genetic basis of index patients with familial hypercholesterolemia in Chinese population: mutation spectrum and genotype-phenotype correlation. *Lipids Health Dis.* 17:252. 10.1186/s12944-018-0900-8 30400955 PMC6220500

[B77] Tada H. Kawashiri M. A. Nomura A. Teramoto R. Hosomichi K. Nohara A. (2018). Oligogenic familial hypercholesterolemia, LDL cholesterol, and coronary artery disease. *J. Clin. Lipidol.* 12 1436–1444. 10.1016/j.jacl.2018.08.006 30241732

[B78] Talmud P. J. Shah S. Whittall R. Futema M. Howard P. Cooper J. A. (2013). Use of low-density lipoprotein cholesterol gene score to distinguish patients with polygenic and monogenic familial hypercholesterolaemia: a case-control study. *Lancet* 381 1293–1301. 10.1016/S0140-6736(12)62127-8 23433573

[B79] Tichý L. Fajkusova L. Zapletalova P. Schwarzova L. Vrablík M. Freiberger T. (2017). Molecular genetic background of an autosomal dominant hypercholesterolemia in the Czech Republic. *Physiol. Res.* 66(Suppl. 1), S47–S54. 10.33549/physiolres.933587 28379029

[B80] Tichý L. Freiberger T. Zapletalová P. Soška V. Ravčuková B. Fajkusová L. (2012). The molecular basis of familial hypercholesterolemia in the Czech Republic: spectrum of LDLR mutations and genotype-phenotype correlations. *Atherosclerosis* 223 401–408. 10.1016/j.atherosclerosis.2012.05.014 22698793

[B81] Timms K. M. Wagner S. Samuels M. E. Forbey K. Goldfine H. Jammulapati S. (2004). A mutation in PCSK9 causing autosomal-dominant hypercholesterolemia in a Utah pedigree. *Hum. Genet*. 114 349–353. 10.1007/s00439-003-1071-9 14727179

[B82] Tveten K. Strøm T. B. Cameron J. Berge K. E. Leren T. P. (2012). Mutations in the SORT1 gene are unlikely to cause autosomal dominant hypercholesterolemia. *Atherosclerosis* 225 370–375. 10.1016/j.atherosclerosis.2012.10.026 23102784

[B83] Vallejo-Vaz A. J. Kondapally Seshasai S. R. Cole D. Hovingh G. K. Kastelein J. J. Mata P. (2015). Familial hypercholesterolaemia: A global call to arms. *Atherosclerosis* 243 257–259. 10.1016/j.atherosclerosis.2015.09.021 26408930

[B84] Vrablik M. Raslova K. Vohnout B. Blaha V. Satny M. Kyselak O. (2018). Real-life LDL-C treatment goals achievement in patients with heterozygous familial hypercholesterolemia in the Czech Republic and Slovakia: results of the PLANET registry. *Atherosclerosis* 277 355–361. 10.1016/j.atherosclerosis.2018.08.008 30270071

[B85] Vrablik M. Zlatohlavek L. Stulc T. Adamkova V. Prusikova M. Schwarzova L. (2014). Statin-associated myopathy: from genetic predisposition to clinical management. *Physiol. Res.* 63(Suppl. 3), S327–S334. 25428737 10.33549/physiolres.932865

[B86] Watts G. F. Ding P. Y. George P. Hagger M. S. Hu M. Lin J. (2016). Translational research for improving the care of familial hypercholesterolemia: the “ten countries study” and beyond. *J. Atheroscler. Thromb*. 23 891–900. 10.5551/jat.35949 27384016 PMC7399296

[B87] Williams R. R. Hasstedt S. J. Wilson D. E. Ash K. O. Yanowitz F. F. Reiber G. E. (1986). Evidence that men with familial hypercholesterolemia can avoid early coronary death. An analysis of 77 gene carriers in four Utah pedigrees. *JAMA* 255 219–224. 3941501

[B88] Yang K. C. Su Y. N. Shew J. Y. Yang K. Y. Tseng W. K. Wu C. C. (2007). LDLR and ApoB are major genetic causes of autosomal dominant hypercholesterolemia in a Taiwanese population. *J. Formos. Med. Assoc*. 106 799–807. 10.1016/S0929-6646(08)60044-3 17964958

[B89] Zaffiri L. Gardner J. Toledo-Pereyra L. H. (2012). History of antibiotics. From salvarsan to cephalosporins. *J. Invest. Surg.* 25 67–77. 10.3109/08941939.2012.664099 22439833

[B90] Zlatohlavek L. Zídkova K. Vrablík M. Haas T. Prusíkova M. Svobodova H. (2008). Lipoprotein(a) and its position among other risk factors of atherosclerosis. *Physiol. Res*. 57 777–783. 18198982 10.33549/physiolres.931133

